# Optimization of eco-friendly ultrasonic probe-assisted extraction from *Stevia rebaudiana* Bertoni using response surface methodology and antioxidant evaluation

**DOI:** 10.1515/biol-2025-1349

**Published:** 2026-06-30

**Authors:** Gizem Yıldırım Baştemur, Reyhan Akpinar, Emir Bora Taşçı, Beyza Pirdal, Nazlı Kılağuz, Furkan Kütükçü, Tuğsan Tenekeci, Merve Boyraz, Sabriye Percin Ozkorucuklu

**Affiliations:** Department of Molecular Biology and Genetics, Faculty of Science, Istanbul University, 34134, Istanbul, Türkiye; Programme of Molecular Biotechnology and Genetics, Institute of Graduate Studies in Science, Istanbul University, 34116, Istanbul, Türkiye; Center for Research and Practice in Biotechnology and Genetic Engineering, Istanbul University, 34134, Istanbul, Türkiye

**Keywords:** *Stevia rebaudiana* Bertoni, response surface methodology, antioxidant, ultrasonic probe-assisted extraction, green HPLC

## Abstract

In this study, an environmentally friendly ultrasonic probe-assisted extraction (UPAE) method was modeled and optimized to enhance the recovery of rebaudioside A, resveratrol, and total phenolic content (TPC) from *Stevia rebaudiana* Bertoni using response surface methodology. A central composite design was applied to investigate the effects of extraction time (1–9 min), ethanol concentration (0–100 %), plant amount (0.1–0.5 g), and ultrasonic frequency (20–28 kHz). Extraction efficiency was evaluated through TPC analysis and quantitative determination of rebaudioside A and resveratrol by high-performance liquid chromatography (HPLC). Green chemistry principles were integrated into extraction and analytical procedures by employing ethanol as a sustainable solvent and eliminating toxic organic modifiers. Optimal conditions were determined as 7 min extraction time, 75 % ethanol, 0.2 g plant material, and 22 kHz frequency. Under these conditions, rebaudioside A, resveratrol, and TPC were obtained as 281.59 μg/mg, 5.74 μg/mg, and 54.17 mg GAE/g, respectively, showing strong agreement with model predictions. The optimized extract exhibited high total flavonoid content and antioxidant capacity as confirmed by DPPH, FRAP, and CUPRAC assays. The proposed UPAE–HPLC approach provides a sustainable and efficient platform for the recovery of natural sweeteners and antioxidant compounds from *Stevia rebaudiana*, with potential applications in food, nutraceutical, and pharmaceutical industries.

## 1 Introduction

*Stevia rebaudiana* Bertoni, commonly known as Stevia, is a perennial plant belonging to the Asteraceae family [1]. This species, which is native to South America, is now cultivated in different geographical regions such as Asia, Europe and North America [2]. Countries such as China, Japan, Korea and Turkey are among the leading producers of Stevia production. Especially the leaves of *Stevia rebaudiana* Bertoni contain phenolic compounds such as flavonoids, alkaloids, and steviol glycosides such as stevioside, rebaudioside A, dulcoside A and steviolbioside which have significant antioxidant and antibacterial properties [3]. These steviol glycosides contribute to the high sweetness level of the plant, being 200 to 300 times sweeter than sucrose, making *Stevia rebaudiana* Bertoni quite popular in the food industry as a non-caloric natural sweetener [4]. Among the approximately 230 species of the genus Stevia, only *Stevia rebaudiana* Bertoni has the capacity to produce these characteristic sweet compounds, making the plant valuable for various industrial and pharmaceutical applications [5].

Conventional extraction methods such as pressing, infusion, maceration, percolation, decoction, Soxhlet extraction, and orbital shaking are commonly applied for the isolation of plant metabolites; however, these techniques generally require long extraction times, high solvent consumption, and may lead to the degradation of thermolabile compounds. Therefore, modern green extraction technologies, including ultrasound-assisted extraction (UAE), microwave-assisted extraction (MAE), pressurized liquid extraction (PLE), subcritical water extraction (SWE), and supercritical fluid extraction (SFE), have gained considerable attention due to their improved extraction efficiency, reduced solvent usage, shorter processing times, and enhanced recovery of bioactive compounds [6], 7]. Among these methods, UPAE is particularly advantageous because acoustic cavitation enhances cell wall disruption and mass transfer, resulting in higher extraction yields under mild operating conditions [8]. These advantages make UPAE an efficient and environmentally friendly approach for the extraction of steviol glycosides and phenolic compounds from *Stevia rebaudiana* extracts [9], 10].

The extraction efficiency of phenolic compounds and steviol glycosides from plants is significantly affected by various process parameters such as extraction time, temperature, type of solvent used and solvent ratio [11]. Therefore, optimizing these parameters is of great importance in terms of obtaining maximum yield, increasing process efficiency, and ensuring the sustainability of the extraction process [12]. Among the various optimization approaches used for this purpose, Response Surface Methodology (RSM) stands out as a powerful statistical method that enables systematic and effective optimization of processes involving a large number of variables and is widely applied in the literature [13]. RSM enables modelling and analysis of multiple independent variables as well as interactions between these variables, allowing the determination of the most suitable conditions that maximize extraction efficiency while reducing process time and resource usage [14], 15].

After the extraction of bioactive compounds of plant origin, the correct qualitative and quantitative analysis of these compounds is of great importance for the reliability of the analytical results. For this purpose, high-performance liquid chromatography (HPLC) method is frequently preferred due to its superior analytical properties such as high resolution, sensitivity, and reproducibility [16], 17]. However, organic solvents widely used in traditional HPLC methods pose various risks to the environment and human health and cause significant hazardous waste production [18]. This situation has increased the interest in environmental sustainability in analytical chemistry and encouraged the development of environmentally friendly HPLC approaches based on green analytical chemistry principles [19]. Green HPLC applications reduce both environmental impact and contribute to the adoption of sustainable analytical methods by enabling the use of less harmful, biodegradable, and economical alternatives instead of toxic and volatile solvents.

Plants rich in phenolic compounds, such as *Stevia rebaudiana* Bertoni, attract attention not only for their natural sweetener potential but also for their significant antioxidant activities, as demonstrated in recent phytochemical and biological evaluation studies [20]. Phenolic compounds play a role in reducing oxidative damage by neutralizing reactive oxygen species thanks to the hydroxyl groups in their structures [21], 22]. Oxidative stress caused by reactive oxygen species is considered to be one of the main factors in the development of many chronic diseases such as cancer, cardiovascular diseases, and neurodegenerative disorders [23]. Therefore, total phenolic content (TPC), total flavonoid content (TFC), and antioxidant activity assays based on different reaction mechanisms, such as DPPH (2,2-diphenyl-1-picrylhydrazyl), FRAP (ferric reducing antioxidant power) and CUPRAC (cupric ion reducing antioxidant capacity) are widely used to evaluate the antioxidant potential of plant extracts [24]. These analyses enable a more comprehensive assessment of the biological potential of extracts by collectively evaluating their phenolic and flavonoid contents, radical scavenging capacity, and reducing power. Since natural antioxidants obtained from plants are evaluated as supportive elements in the prevention of these diseases, the rich phenolic content of *Stevia rebaudiana* Bertoni makes it a valuable biological resource not only in the food sector but also in pharmaceutical and nutraceutical applications [25], 26].

This study was carried out to develop and optimize extraction conditions for increasing the bioactive compound content in extracts obtained from *Stevia rebaudiana* Bertoni. For this purpose, ultrasonic probe-assisted extraction (UPAE) method was preferred in accordance with environmental sustainability principles and statistical modelling was performed with the central composite design (CCD) approach to determine the optimum extraction conditions. The effects of process parameters such as extraction time, ethanol content, plant amount, and ultrasonic frequency were evaluated using two different analytical methods. The first was TPC analysis; the other was an environmentally friendly HPLC method, adhering to green analytical chemistry principles, for the quantitative determination of rebaudioside A and resveratrol. Total flavonoid content and antioxidant activities of the extract obtained under optimum conditions were analysed by DPPH, FRAP and CUPRAC methods.

## 2 Materials and methods

### 2.1 Reagents and chemicals

Resveratrol (≥98 %), rebaudioside A (≥96 %), Folin–Ciocalteu reagent, 2,2-diphenyl-1-picrylhydrazyl (DPPH), sodium carbonate, sodium acetate, aluminum chloride, TPTZ (2,4,6-tripyridyl-s-triazine), iron(III) chloride, copper(II) chloride dihydrate, neocuproine, and standard reference compounds including gallic acid (≥99 %), rutin (≥94 %), trolox (≥97 %), and ascorbic acid (≥99 %), were all purchased from Sigma-Aldrich (St. Louis, MO, USA). HPLC-grade ethanol and orthophosphoric acid were obtained from Merck Specialities Pvt. Ltd (Darmstadt, Germany). All stock solutions were prepared using ultrapure water obtained from a Milli-Q system (Millipore, Bedford, MA, USA, resistivity ≥18.2 MΩ cm). Prior to analysis, the mobile phase, standard solutions, and *Stevia rebaudiana* Bertoni extracts were filtered through 0.45 μm PTFE membrane filters to remove particulates.

### 2.2 Plant material and extraction procedure

*Stevia rebaudiana* Bertoni plant used in the study was supplied by Silivri Municipality Agricultural Production and Research Center (TÜRAM, Istanbul, Turkey). The plant material was air-dried at room temperature and then ground to obtain a homogeneous powder. UPAE was performed using a Sonoplus HD 4100 (Bandelin electronic GmbH & Co. KG, Berlin, Germany). For each experimental run, the specified amount of plant material (0.1–0.5 g, according to the experimental design) was mixed with 5 mL of ethanol–water solvent system at the desired ethanol ratio (0–100 %, v/v). Therefore, the solid-to-liquid ratio varied between 1:50 and 1:10 (g/mL), depending on the plant material amount. The extractions were carried out according to the conditions generated by the response surface methodology (RSM), including extraction time (1–9 min) and ultrasonic frequency (20–28 kHz). During sonication, the probe was immersed directly into the extraction mixture, and temperature was monitored to avoid excessive heating. After extraction, the mixtures were centrifuged at 9,500 rpm for 15 min, and the supernatants were filtered through 0.45 µm syringe filters. The solvent was removed using a vacuum evaporator, and the dried extracts were re-dissolved in ethanol and stored at 4 °C until analysis. The extraction efficiency was evaluated according to the ratio of the crude extract obtained after extraction to the initial sample amount, as described by Karimipour et al. [27]. The extraction yield was calculated using the equation below and the obtained value was calculated as 27.2 %.

$$\text{Extraction yield }\left(\%\right)=\frac{\text{the}\;\text{mass}\;\text{of}\;\text{crude}\;\text{extract}\;}{\;\text{the}\;\text{mass}\;\text{of}\;\text{the}\;\text{sample}}\times 100$$

### 2.3 Experimental design

Response surface methodology includes different experimental design approaches, such as central composite design and Box–Behnken Design [28]. In this study, Central Composite Design was preferred for the optimization process. The results of the experimental conditions designed with central composite design were also evaluated with design expert software. Extraction time (*X*_1_), ethanol ratio (*X*_2_), plant amount (*X*_3_) and ultrasonic frequency (*X*_4_) were selected as independent variables at 5 different levels (−2, −1, 0, +1, +2) (Table 1) and the design consisted of 30 experimental runs. The dependent variables were defined as rebaudioside A (*Y*_1_), resveratrol (*Y*_2_), and total phenolic content (*Y*_3_). A second-order polynomial model was used to describe the relationship between the independent variables and the responses.

**Table 1:** Value range of central composite design with four independent variables.

Factors	Symbol of the variables	Levels				
		−2	−1	0	+1	+2
Extraction time	*X*_1_	1	3	5	7	9
Ethanol ratio	*X*_2_	0	25	50	75	100
Plant amount	*X*_3_	0.1	0.2	0.3	0.4	0.5
Ultrasonic frequency	*X*_4_	20	22	24	26	28

The extraction parameters and their respective ranges were selected based on preliminary experiments, literature reports on ultrasonic-assisted extraction of phenolics and steviol glycosides, and the operational limits of the ultrasonic probe system. The selected levels were defined to adequately model linear, interaction, and quadratic effects within the central composite design framework. To ensure controlled cavitation intensity and reduce model complexity, the ultrasonic amplitude was kept constant at 245 µm throughout all experiments. Extraction temperature was monitored during the experiments and remained within a narrow range; therefore, it was not included as an independent optimization parameter.

### 2.4 HPLC analysis

**Figure 1:**
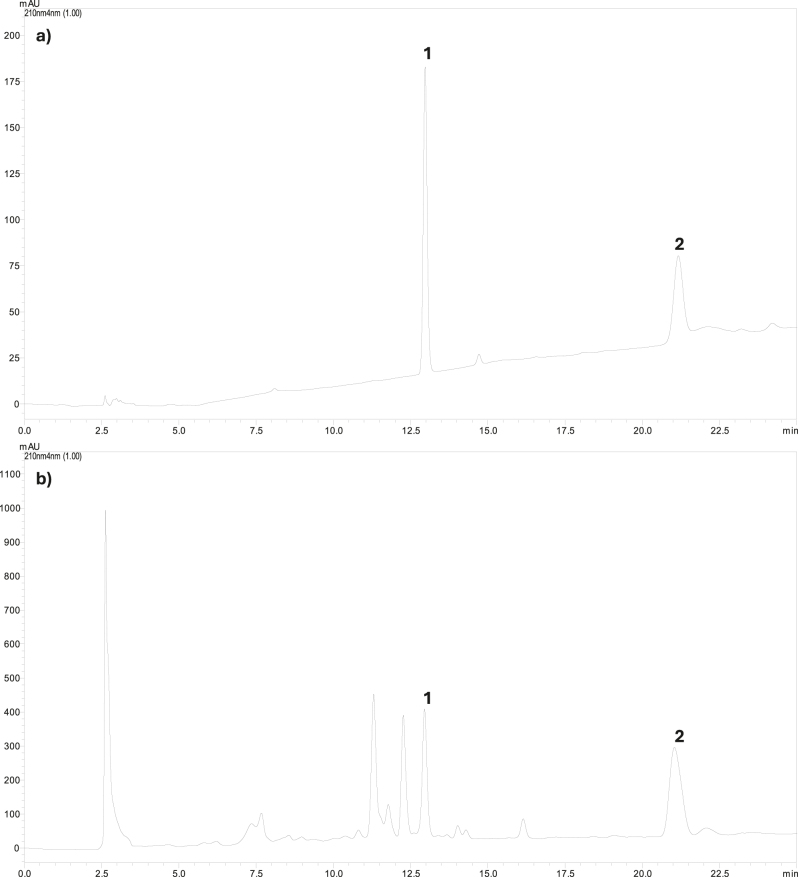
(a) Standard chromatogram of resveratrol (1; Rt = 12.97) and rebaudioside A (2; Rt = 21.16). (b) Chromatogram of the extract at optimum extraction conditions.

Quantitative analysis of resveratrol and rebaudioside A was performed using Shimadzu HPLC system (Shimadzu Corporation, Kyoto, Japan) equipped with an LC-10AD VP pump, CTO-10AS column oven, DGU-20A degasser and SPD-M20A diode array detector (DAD). The chromatographic separation was achieved on an Agilent ZORBAX Eclipse Plus C18 column (250 mm × 4.6 mm, 5 µm). The mobile phases consisted of (A) deionized water containing 0.5 % orthophosphoric acid and (B) ethanol. The gradient program was as follows: 0–12 min, 10–40 % B; 12–22 min, 40–50 % B; 22–25 min, 50–10 % B. The flow rate was set to 1.0 mL/min, the column temperature maintained at 25 °C, the injection volume at 20 µL, and detection wavelength at 210 nm. Ethanol was selected as the organic phase to replace acetonitrile and methanol, minimizing environmental hazards and improving the greenness of the analytical procedure. Calibration curves were constructed by plotting peak areas on the *Y*-axis against concentration values on the *X*-axis. The concentration ranges were set as 750–2,500 μg/mL for rebaudioside A and 1–25 μg/mL for resveratrol. The *R* ^2^ values calculated from the calibration curves constructed for rebaudioside A and resveratrol were found to be 0.9999 and 0.9995, respectively, with regression equations of *y* = 4,727.4*x* + 287,745 and *y* = 1,381,06*x* – 65,652. Chromatograms of the standards (rebaudioside A and resveratrol) and the extract obtained under optimum extraction conditions are presented in Figure 1. Limit of detection (LOD) and limit of quantification (LOQ) are common parameters for evaluating the sensitivity of analytical methods [29]. LOD and LOQ values for rebaudioside A were determined as 29.31 μg/mL and 97.70 μg/mL, respectively, and for resveratrol as 0.02 μg/mL and 0.07 μg/mL. The amounts of rebaudioside A and resveratrol in the plant extracts were calculated using the obtained regression equations, and the results were expressed as micrograms of compound per milligram of dry extract (µg/mg dry extract).

### 2.5 Determination of total phenolic content

The total phenolic content (TPC) of the extracts was determined using the Folin–Ciocalteu colorimetric method, which is based on the reduction of the Folin–Ciocalteu reagent by phenolic compounds under alkaline conditions, resulting in the formation of a blue chromophore. In this context, the method was applied according to the procedure previously described by Raghu and Velayudhannair [30] with some modifications. The analysis was performed using a calibration curve obtained from seven different gallic acid concentrations ranging from 0.25 μg/mL to 250 μg/mL (*R* ^2^ = 0.9996). To perform the assay, firstly, 100 µL of the sample (standard, extract, blank) solution and 200 µL of 10 % Folin reagent (v/v) were mixed and vortexed. After vortexing, the mixture was incubated at room temperature for 5 min. Subsequently, 800 µL of 2 % Na_2_CO_3_ was added and incubated on a shaker for 90 min. After incubation, the absorbance of the reaction mixture was measured at 765 nm using a BioTek Instruments spectrophotometer (BioTek Instruments Inc., Winooski, VT, USA). To ensure analytical reliability, each measurement was performed in triplicate and repeated on two separate days. The total phenolic content was expressed as milligrams of gallic acid equivalents per gram of dry extract weight (mg GAE/g), and the results were reported as mean values with their corresponding standard deviations (mean ± SD). These values represent the concentration of phenolic and flavonoid compounds within the extract itself rather than the overall extraction yield relative to the initial plant material.

### 2.6 Determination of total flavonoid content

Total flavonoid content (TFC) was determined by modifying the colorimetric method reported by Wingren et al. [31]. For each reaction, 100 μL of sample solution was mixed with 300 μL ethanol, 20 μL of 10 % (w/v) aluminum chloride, 20 μL of 1 M potassium acetate, and 560 μL of distilled water. The mixture was incubated for 20 min at room temperature, and then the absorbance was recorded at 415 nm. A calibration curve was constructed using rutin as the reference compound, and total flavonoid content were expressed as milligrams of rutin equivalents per gram of dry extract (mg RE/g). These values represent the concentration of phenolic and flavonoid compounds within the extract itself rather than the overall extraction yield relative to the initial plant material. All analyses were carried out in triplicate.

### 2.7 Antioxidant activity assays

#### 2.7.1 DPPH (1-1-diphenyl 2-picryl hydrazyl) radical scavenging activity

The DPPH (2,2-diphenyl-1-picrylhydrazyl) assay is a widely recognized method for evaluating antioxidant activity based on the capacity of compounds to donate hydrogen atoms or electrons to neutralize free radicals. Originally developed by Bao et al., this method employs the stable nitrogen-centered free radical DPPH, which exhibits a deep violet color due to its unpaired electron [32]. When antioxidant molecules capable of donating hydrogen atoms interact with DPPH radicals, the DPPH molecule is reduced, leading to a noticeable change in color from deep violet to yellow. This decolorization is quantitatively measured by a decrease in absorbance at 517 nm, which represents the maximum absorption wavelength of DPPH in ethanol [33]. The degree of discoloration correlates with the radical-scavenging activity of the test sample, where higher inhibition percentages reflect greater antioxidant potential. Based on this principle, in this study, DPPH radical scavenging activity was determined with using the method described by Demirag [20]. In this method, 100 µL of 0.2 mM DPPH solution was mixed with 100 µL of sample solution (standards, extract and blank) in a well of 96 plates. l-ascorbic acid and trolox (0.13–25 μg/mL) were used as positive controls; distilled water and methanol were used as blanks. The mixture was shaken for 30 min in the dark at room temperature. After incubation, the absorbance was read against the blank at 517 nm using a spectrophotometer. All measurements were performed in triplicate at two different times. The DPPH radical scavenging activity was calculated as a percentage according to the following equation:

$$\text{DPPH radical scavenging activity }\left(\%\right)=\left(\frac{A_{\text{blank}}-A_{\text{sample}}}{A_{\text{blank}}}\right)\times 100$$

where: *A*_blank_ is the absorbance of the blank, *A*_sample_ is the absorbance of the sample.

#### 2.7.2 FRAP assay (ferric reducing antioxidant power)

The Ferric Reducing Antioxidant Power (FRAP) assay is a commonly used method for evaluating the antioxidant capacity of various substances, including plant extracts, foods, and biological samples. Developed by Benzie and Strain, the assay is based on the reduction of ferric ions (Fe^3+^) to ferrous ions (Fe^2+^) by antioxidants in an acidic environment, resulting in the formation of a blue-colored ferrous–tripyridyltriazine (Fe^2+^–TPTZ) complex. This complex can be quantitatively measured by its absorbance at 593 nm. Accordingly, the FRAP assay was performed in this study according to the method described by Benzie and Strain, with minor modifications [34]. The FRAP reagent consisted of 0.3 M acetate buffer (pH 3.6), 10 mM TPTZ in 40 mM HCl, and 20 mM FeCl_3_·6H_2_O mixed in a 10:1:1 ratio. During the analysis, 280 µL of FRAP reagent and 20 µL of sample (standard, extract or blank) were added to each well of the 96-well microliter plate and the mixture was incubated at approximately 37 °C for 30 min. After incubation, the absorbance of the reaction mixture was measured at 593 nm wavelength. A calibration curve was created with trolox standard solutions and the antioxidant capacity was expressed as µmol Trolox equivalent per gram of dry extract (µmol TE/g). All measurements were carried out at two different time points, each with three replicates.

#### 2.7.3 CUPRAC assay (cupric ion reducing antioxidant capacity)

The CUPRAC method is based on the reduction of a cupric-neocuproine (Cu(II)-Nc) complex to the cuprous (Cu(I))-neocuproine chelate by antioxidant compounds under near-neutral pH conditions. This redox reaction results in the generation of an orange-colored complex exhibiting a maximum absorbance at 450 nm, enabling its detection and quantification through spectrophotometric analysis. In this study, the CUPRAC assay was performed according to Apak et al. [35]. In this method, which was carried out to determine antioxidant capacity, the mixture required for the analysis consisted of 250 µL of 10 mM copper(II) chloride dihydrate (CuCl_2_ 2H_2_O), 250 µL of 7.5 mM neocuproine, 250 µL of 1.0 M ammonium acetate (NH_4_Ac) buffer solution (pH 7.0), 25 µL of sample solution (extract, standard and blank) and 250 µL of distilled water. These components were transferred to each well of a 48-well microplate, respectively, and a total reaction mixture volume of 1,025 µL was obtained. After incubation for 60 min in the dark at room temperature, absorbance was recorded at 450 nm. A calibration curve was created using trolox standard solutions and the molar absorptivity coefficient (*ε*) of trolox was calculated by plotting the absorbance values ​​obtained against the relevant concentrations. Total antioxidant capacity (TAC) was expressed as mmol Trolox equivalent per gram of extract, and EC_50_ values were calculated as the concentration required to reach half-maximal absorbance.

### 2.8 Statistical analysis

The significance level of the model was subjected to Analysis of Variance (ANOVA) in order to establish and define the relationships between the responses and the variables. The optimization process was carried out using Response Surface Methodology (RSM) in Design-Expert 12 software. In statistical evaluations, the significance level was accepted as *p* < 0.05. The fit and adequacy of the polynomial model equation were examined through the coefficient of determination (*R* ^2^, adjusted *R* ^2^, and predicted *R* ^2^) values; the significance of the regression coefficients was evaluated using the *F* test. In addition, the coefficient of variation (CV, %) showing the reliability of the model was calculated and taken into account as a criterion of acceptability. All analyses were conducted in three independent and parallel experiments, and the results were expressed as mean ± standard deviation (SD).

**Research ethics:** Not applicable.

**Informed consent:** Not applicable.

## 3 Results and discussion

### 3.1 Model fitting and statistical analysis (ANOVA)

The effects of four extraction parameters (extraction time, ethanol ratio, plant amount, and ultrasonic frequency) on the amounts of rebaudioside A and resveratrol and total phenolic content were evaluated. The responses of these parameters under 30 independent conditions are summarized in Table 2.

**Table 2:** Experimental values of rebaudioside A (RebA), resveratrol, and TPC at various levels of input variables as suggested by experimental design.

Extraction parameters					Response		
Run	Extraction time (min) (*X*_1_)	Ethanol ratio (%) (*X*_2_)	Plant amount (g) (*X*_3_)	Ultrasonic frequency (kHz) (*X*_4_)	RebA (µg/mg) ± SD (*Y*_1_)	Resveratrol (µg/mg) ± SD (*Y*_2_)	TPC (mg GAE/g) ± SD (*Y*_3_)
1	3	25	0.2	22	220.08 ± 2.17	3.556 ± 0.06	29.56 ± 1.13
2	3	75	0.4	22	252.82 ± 8.41	5.175 ± 0.13	45.79 ± 1.84
3	5	50	0.3	24	186.02 ± 0.61	4.010 ± 0.01	32.44 ± 0.81
4	7	25	0.4	26	164.61 ± 0.47	2.956 ± 0.01	24.33 ± 0.77
5	3	25	0.4	26	177.68 ± 1.53	3.250 ± 0.06	34.80 ± 2.65
6	5	50	0.1	24	200.42 ± 0.89	4.386 ± 0.03	42.25 ± 3.25
7	7	25	0.4	22	170.12 ± 1.05	2.945 ± 0.02	25.90 ±0.49
8	**7**	**75**	**0.2**	**22**	**281.59** ± **0.62**	**5.746** ± **0.02**	**53.83** ± **1.00**
9	3	75	0.2	22	263.96 ± 2.20	5.361 ± 0.05	49.39 ± 3.33
10	5	50	0.3	20	210.47 ± 1.29	4.396 ± 0.04	35.67 ± 1.11
11	3	75	0.2	26	228.35 ± 0.38	4.940 ± 0.02	35.00 ± 0.81
12	7	25	0.2	26	170.85 ± 0.71	2.990 ± 0.02	25.04 ± 0.73
13	3	25	0.2	26	159.34 ± 0.64	2.758 ± 0.01	22.41 ± 0.66
14	5	50	0.3	28	209.52 ± 0.74	4.455 ± 0.02	33.78 ± 0.83
15	5	0	0.3	24	169.02 ± 1.29	2.131 ± 0.02	20.65 ± 0.18
16	5	50	0.3	24	167.97 ± 1.57	3.616 ± 0.02	33.88 ± 0.79
17	5	50	0.3	24	210.40 ± 0.26	4.505 ± 0.01	32.48 ± 0.84
18	9	50	0.3	24	203.10 ± 1.44	4.192 ± 0.03	36.22 ± 0.77
19	5	50	0.5	24	185.29 ± 1.88	3.871 ± 0.02	33.04 ± 0.25
20	7	75	0.4	22	265.64 ± 0.18	5.445 ± 0.01	46.02 ± 2.17
21	7	75	0.4	26	250.03 ± 0.17	5.052 ± 0.03	47.92 ± 5.88
22	5	100	0.3	24	298.73 ± 1.43	5.046 ± 0.03	51.58 ± 1.92
23	7	75	0.2	26	238.45 ± 2.89	5.051 ± 0.05	47.06 ± 0.61
24	3	75	0.4	26	231.20 ± 1.50	4.634 ± 0.04	46.68 ± 0.79
25	5	50	0.3	24	220.90 ± 2.20	4.795 ± 0.08	36.89 ± 0.54
26	3	25	0.4	22	152.08 ± 1.31	2.657 ± 0.03	23.66 ± 1.16
27	5	50	0.3	24	208.98 ±0.32	4.432 ± 0.02	33.16 ± 1.00
28	1	50	0.3	24	211.62 ± 0.69	4.617 ± 0.02	35.70 ± 3.04
29	7	25	0.2	22	210.96 ± 0.91	3.54 ± 0.02	33.30 ± 0.74
30	5	50	0.3	24	223.03 ± 0.53	4.75 ± 0.04	41.71 ± 1.63

The optimum extraction conditions have been highlighted in bold.

The model’s suitability is determined by the coefficient of determination (*R* ^2^), which takes values ​​between 0 and 1. An *R* ^2^ value close to 1 is expected, indicating that the model is compatible with the actual data [12], 36], 37]. In other words, for a model to be considered statistically significant, the difference between the estimated *R* ^2^ and the adjusted *R* ^2^ should be less than 0.2 [12]. The resulting models demonstrated robust correlation coefficients *R*_RebA_^2^ = 0.90, *R*_Resveratrol_^2^ = 0.91, and *R*_TPC_^2^ = 0.93. The predicted values of *R* ^2^ that is *R*_RebA_^2^ = 0.70, *R*_Resveratrol_^2^ = 0.72, and *R*_TPC_^2^ = 0.73, and the adjusted values of *R* ^2^ that is *R*_RebA_^2^ = 0.81, *R*_Resveratrol_^2^ = 0.83, and *R*_TPC_^2^ = 0.86. The result shows that the approximately 0.2 difference between the adjusted and predicted *R* ^2^ values indicates an acceptable level of agreement, suggesting that the models possess good predictive ability and are not overfitted.

The coefficient of variation (CV, %) is expressed as the standard deviation relative to the mean in percentage terms. Generally, for the model to be considered adequate, the CV should be <10 % [38]. Coefficients of variation C.V_Reb A_% = 8.00, C.V_Resveratrol_% = 9.37, and C.V_TPC_% = 9.37 confirming their adherence to the specified criteria [39], [40], [41]. Effects with a *p* value greater than 0.05 were considered insignificant. It is stated that if the lack of fit value determined by repeated calculation of the center points is >0.05, it is statistically insignificant and the error is insignificant. Another factor used to evaluate the model is the *F* value. The *F* value is considered insignificant for model inadequacy, but significant for the regression model. In addition, the validity of the models was further confirmed through lack of fit analysis, where an insignificant p-value greater than 0.05 indicated a good fit of the models to the actual data. Consequently, all models showed lack of fit values that were not statistically significant (*p* > 0.05). A large *F*-value and a small *p*-value indicate that the independent variables significantly influence the respective response variables [42]. Thus, among the independent variables listed in Table 3, only ethanol ratio (*p* < 0.001) with the highest *F*-values observed: 104.09 for rebaudioside A, 138.72 for resveratrol, and 164.88 for TPC. This finding suggests that ethanol ratio plays a dominant role in optimizing extraction efficiency, potentially due to its effect on solubilizing flavonoid compounds.

**Table 3:** Result of analysis of variance (ANOVA) of rebaudioside A, resveratrol, and TPC.

Source	Rebaudioside A				Resveratrol				TPC			
	d.f.	Mean square	*F*-value	*p*-value	d.f.	Mean square	*F*-value	*p*-value	d.f.	Mean square	*F*-value	*p*-value
Model	14	2,642.40	9.23	<0.0001	14	1.68	10.97	<0.0001	14	160.89	13.70	<0.0001
*X*_1_-extraction time	1	102.95	0.36	0.56	1	0.01	0.08	0.78	1	8.43	0.72	0.41
*X*_2_-ethanol ratio	1	29,801.71	104.09	<0.0001	1	21.24	138.72	<0.0001	1	1936.75	164.88	<0.0001
*X*_3_-plant amount	1	812.82	2.84	0.11	1	0.34	2.23	0.16	1	18.08	1.54	0.23
*X*_4_-ultrasonic frequency	1	1,643.98	5.74	0.03	1	0.30	1.95	0.18	1	32.41	2.76	0.12
Residual	15	286.32	0.42	0.89	15	0.15		0.77	15	11.75		
Lack of fit	10	196.72			10	0.13	0.60		10	10.92	0.82	0.64
Pure error	5				5	0.21			5	13.40		
Collected total	29				29				29			
*R* ^2^	0.90				0.91				0.93			
Adjusted *R* ^2^	0.81				0.83				0.86			
Predicted *R* ^2^	0.70				0.72				0.73			

Second-degree polynomial equations were used to model the extraction efficiencies of the dependent variables (Equations (1)–(3)). These equations provide a mathematical framework to describe the relationships between independent variables and extracted components, facilitating a comprehensive analysis of the extraction process.

(1) $$Y1=+202.88+2.07X_{1}+35.24X_{2}–5.82X_{3}–8.28X_{4}+3.25\;X_{1}X_{2}+0.4064\;X_{1}X_{3}–0.7495\;X_{1}X_{4}+5.25X_{2}X_{3}+2.20X_{2}X_{4}+10.15X_{3}X_{4}+1.76\;X_{1}^{2}+8.39X_{2}^{2}–1.87X_{3}^{2}+2.42X_{4}^{2}$$

(2) $$Y2=+4.35+0.0228X_{1}+0.9408X_{2}–0.1192X_{3}–0.1116X_{4}+0.0607\;X_{1}X_{2}–0.0019\;X_{1}X_{3}–0.0290\;X_{1}X_{4}+0.0155X_{2}X_{3}–0.0815X_{2}X_{4}+0.1335X_{3}X_{4}+0.0117X_{1}^{2}–0.1922X_{2}^{2}–0.0572X_{3}^{2}+0.0171\;X_{4}^{2}$$

(3) $$Y3=+35.31+0.5927X_{1}+8.98X_{2}–0.8680X_{3}-0.2156X_{4}+1.17X_{1}X_{2}–1.85X_{1}X_{3}–0.02491X_{1}\;X_{4}+0.1904X_{2}X_{3}–0.7124X_{2}X_{4}+3.06X_{3}X_{4}+0.4825X_{1}^{2}–0.3499X_{2}^{2}+0.7362X_{3}^{2}+0.0012X_{4}^{2}$$

*According to the formula, the negative coefficient variables for *Y*_1_, *Y*_2_, and *Y*_3_ models suggest adverse effects on the extraction of rebaudioside A, resveratrol and total phenolic content, on the other hand, the positive coefficient variables for *Y*_1_, *Y*_2_, and *Y*_3_ models suggest the favourable effects on the extraction of rebaudioside A, resveratrol and total phenolic content.

### 3.2 Effects of the extraction parameters on the quantification of rebaudioside A

**Figure 2:**
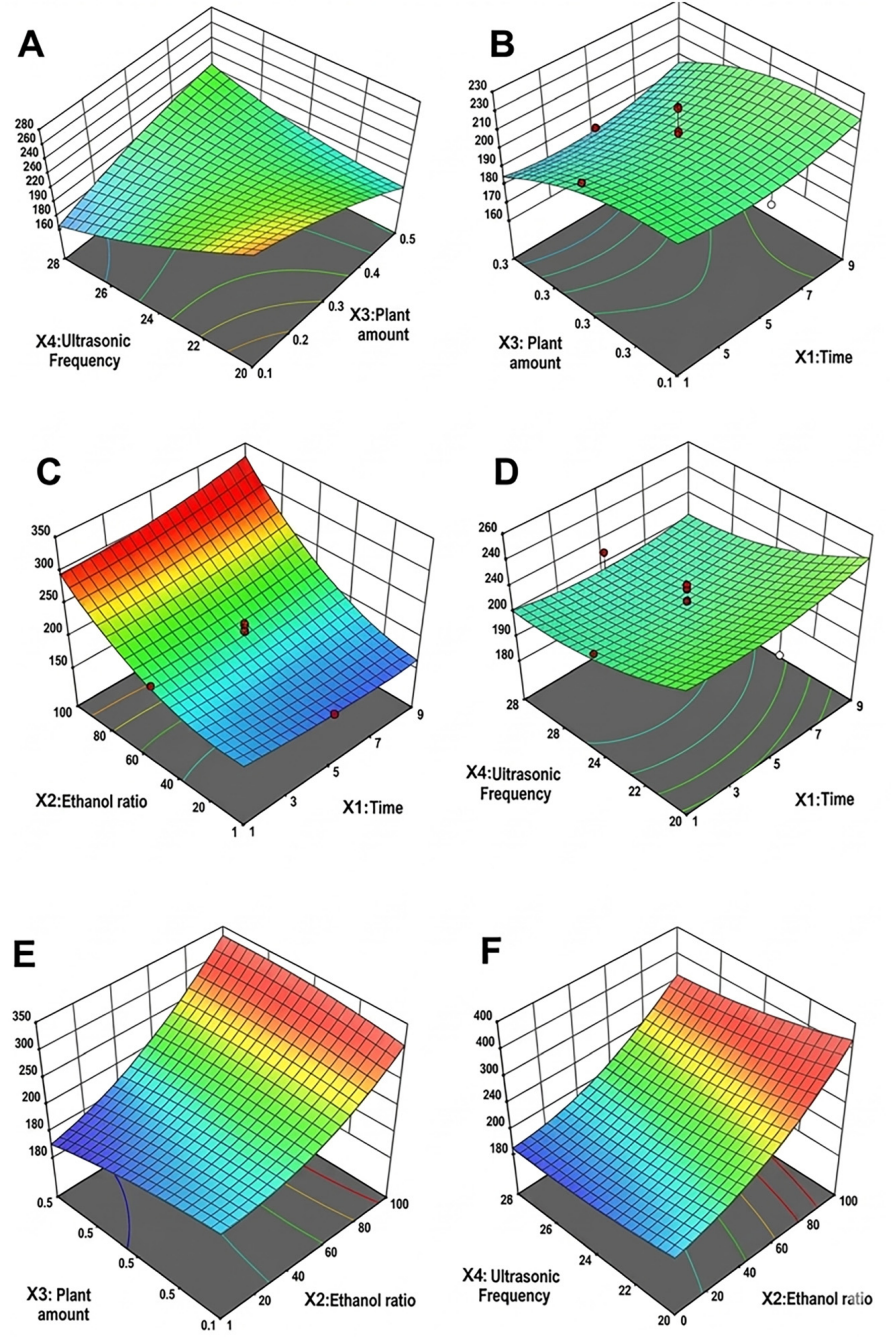
3D and contour response surface plots of rebaudioside A showing the effects of extraction parameters.

Figure 2 shows three-dimensional (3D) surface and contour plots illustrating the interactive effects of various extraction parameters on the yield of rebaudioside A; each plot shows the effect of two independent variables, while the other two are held constant. Among the tested parameters, the ethanol ratio was determined to be the most significant factor affecting the extraction yield of rebaudioside A. As seen in Figure 2C, 2E, and 2F, increasing the ethanol ratio resulted in a significant increase in yield, ranging from 152.08 mg/g to 298.73 mg/g. This finding was also supported by the ANOVA results (Table 3), which confirmed its statistical significance (*p* < 0.001). Furthermore, although methanol and glycerol are cited as effective solvents in the literature, our findings demonstrate that ethanol offers superior extraction efficiency, consistent with environmentally friendly practices [43], 44].

In contrast, as shown in Figure 2B–2D, the extraction time showed statistically insignificant effect (*p* > 0.05), indicating minimal contribution to rebaudioside A yield in the tested ranges. As seen in Figure 2A, 2D, and 2F, the effect of ultrasonic frequency was found to be marginally significant (*F* = 5.74; *p* = 0.0300). However, unlike many previous studies that focused on constant ultrasonic power, this study offered a new perspective by emphasizing the role of ultrasonic frequency (22 kHz). As previously reported, higher frequencies weaken the cavitation effect, limiting cell wall disruption, leading to a decrease in extraction efficiency [45]. This phenomenon is clearly reflected in Figure 2A. The inverse relationship between plant amount and Rebaudioside A yield is clearly shown in Figures 2A, 2B and 2E and supports previous studies [46] showing that higher solvent volumes can reduce extraction efficiency by diluting active compound concentrations.

### 3.3 Effects of the extraction parameters on the quantification of resveratrol

**Figure 3:**
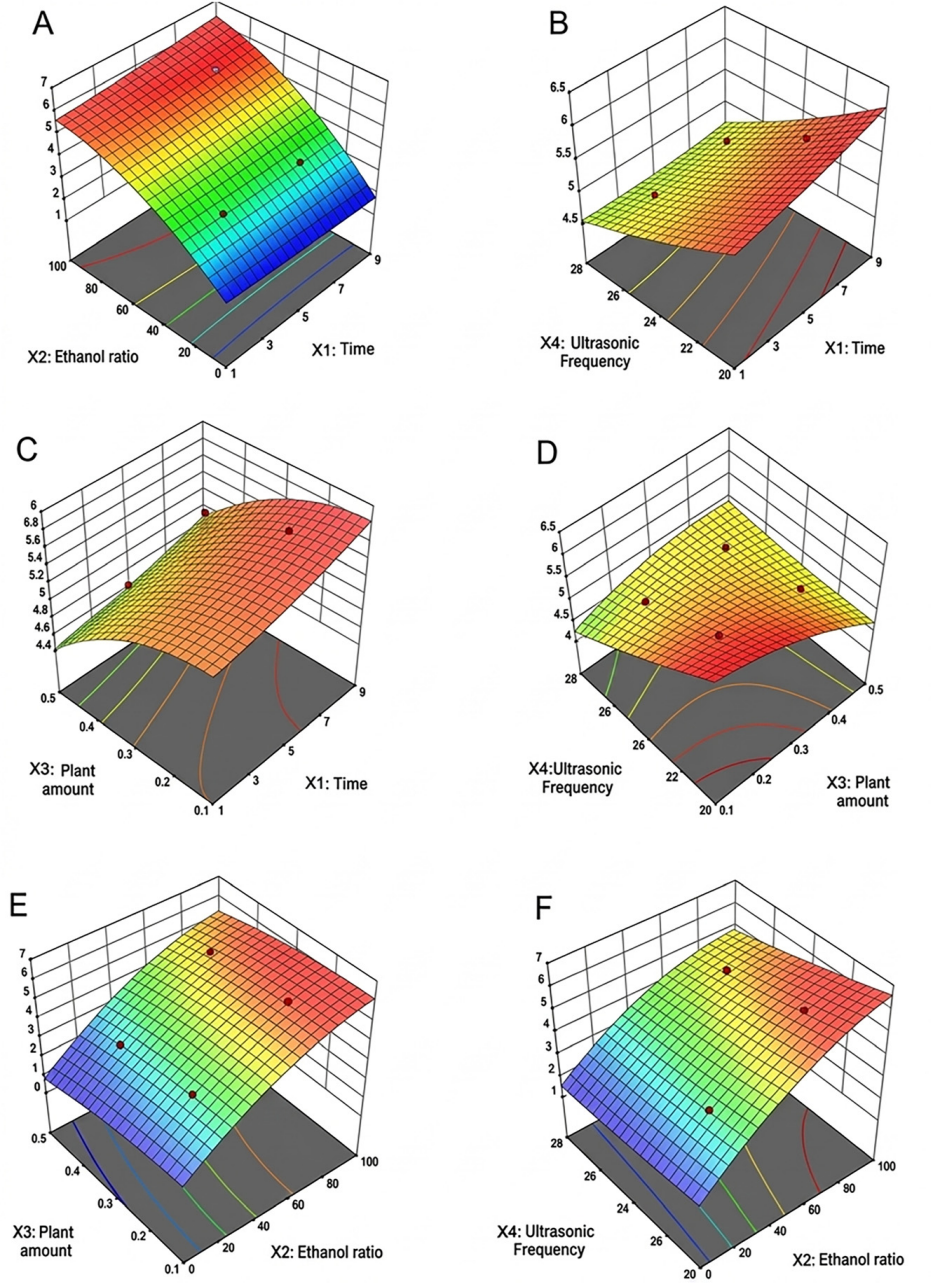
3D and contour response surface plots of resveratrol showing the effects of extraction parameters.

Figure 3 presents three-dimensional (3D) surface and contour plots illustrating the interactive effects of extraction parameters on the resveratrol yield. The ethanol ratio significantly influenced resveratrol yield, as supported by ANOVA results (*p* < 0.0001). However, unlike rebaudioside A, the increase in resveratrol yield with increasing ethanol ratio reached a peak and then decreased between 75 % and 100 % ethanol ratio as seen in Figure 3A, 3E and 3F. This decrease is likely due to the reduced polarity at high ethanol ratio, which limits the solubility and diffusion of resveratrol as a phenolic compound, thereby decreasing extraction efficiency. This observation is consistent with literature findings that report an optimal ethanol ratio for phenolic compound extraction around 70 % [47], [48], [49].

In Figure 3A–3C, the effect of extraction time appeared to influence resveratrol yield. ANOVA results indicate this effect was not statistically significant (*p* > 0.05), although the impact varied across different parameter combinations. This suggests that extended extraction promotes mass transfer and phenolic release, but care must be taken to avoid degradation due to thermal effects. In this study, no adverse effects were observed within the time period tested (up to 9 min), which is consistent with previous findings that short to moderate extraction durations can enhance extraction yield without compromising phenolic stability [47]. Changing the plant amount between 0.1 and 0.5 g caused the solid-to-liquid ratio to vary from 1:50 to 1:10 (g/mL) due to the fixed solvent volume. Therefore, the observed extraction behavior may reflect not only the direct effect of plant amount but also the influence of changes in the solid-to-liquid ratio on mass transfer efficiency, solvent capacity, and solute diffusion. In particular, increasing the plant mass at a fixed solvent volume may limit extraction efficiency due to the limited extraction capacity of the solvent, reduced plant matrix–solvent contact, and lower diffusion efficiency. The effect of plant amount is shown in Figure 3C–3E. These plots indicate that an initial increase in plant amount could enhance resveratrol yield, which may be attributed to the presence of a higher amount of extractable compounds in the extraction medium and improved mass transfer under an appropriate solid-to-liquid ratio. However, a further increase in plant amount led to a plateau or slight decrease in yield. This may be associated with limited solvent capacity under a fixed solvent volume, reduced solute–solvent interactions, and decreased diffusion efficiency. Figure 3B, 3D, and 3F demonstrate the role of ultrasonic frequency in resveratrol extraction. Even though ANOVA results indicate this effect was statistically significant (*p* < 0.0001), the results reveal that higher frequencies tend to reduce resveratrol yield, likely due to lower cavitation intensity and the potential for oxidative degradation induced by excessive ultrasonic energy. This trend supports previous studies suggesting that while ultrasound enhances extraction through cavitation, overly high frequencies may compromise phenolic integrity [50]. In conclusion, resveratrol extraction is influenced by a complex interplay of process parameters, with optimal ethanol ratio (∼75 %), moderate extraction time, and balanced plant amount ratios being critical for maximizing yield without degradation.

### 3.4 Effects of the extraction parameters on the TPC

**Figure 4:**
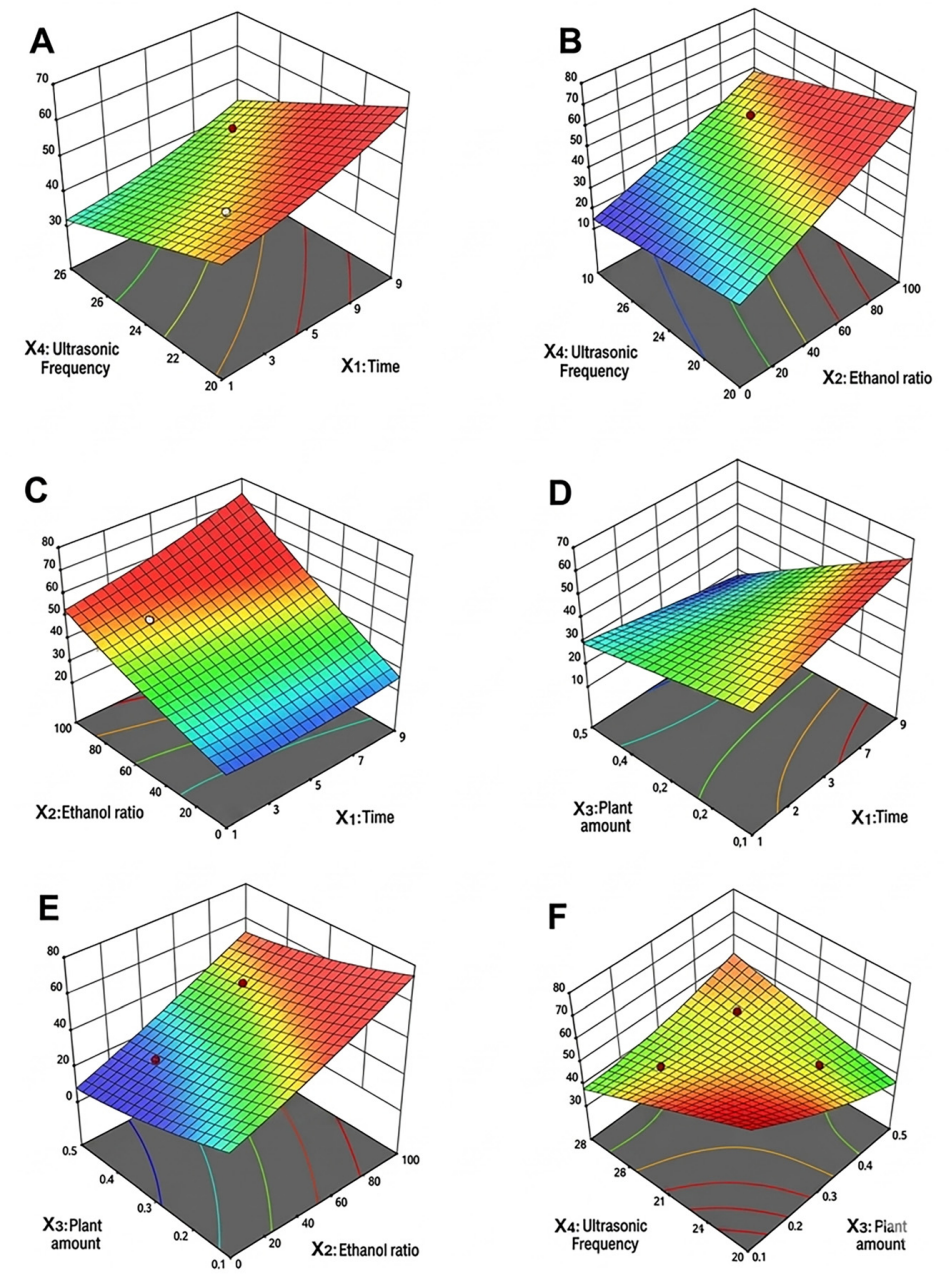
3D and contour response surface plots of TPC showing the effects of extraction parameters.

Three-dimensional (3D) surface and contour plots illustrating the interactive effects of different extraction parameters on the total phenolic content are presented in Figure 4. According to the ANOVA results (Table 3), ethanol ratio had the most dominant and statistically significant effect on TPC (*p* < 0.0001). As seen in Figures 4B, 4C and 4E, TPC remained at levels of approximately 20–25 mg GAE/g at low ethanol ratios (0–25 %), while it increased to over 50 mg GAE/g in the 75–100 % ethanol range. This aligns with previous studies indicating that ethanol enhances the extraction of bioactive compounds due to its ability to disrupt plant cell membranes and facilitate solubilisation [9], 51]. While extraction time is shown in Figure 4A, 4C, and 4D depicted a positive trend in the graphical analysis, it was not found to be statistically significant (*p* > 0.05), though other studies have observed such effects [49]. Figure 4D–F reveal that the plant amount had a comparatively lower impact. Nonetheless, increasing the plant amount beyond an optimal level was associated with a slight decrease in total phenolic content. This trend may be attributed to the over-dilution of active compounds and changes in mass transfer gradients, which reduce extraction efficiency [52]. Additionally, an increased plant amount may disturb extraction equilibrium and affect the ultrasound energy distribution across the solvent volume [53]. The effect of ultrasonic frequency is shown in Figure 4A, 4B, and 4F. Although total phenolic contents were generally higher at lower frequencies (22–26 kHz), these differences were not statistically significant according to the ANOVA results. This observation is consistent with prior findings indicating that higher ultrasonic frequencies may reduce the total amount of phenolic substances due to oxidative degradation mechanisms, although limited data exists on this phenomenon [54].

### 3.5 Optimisation and model verification

The optimal conditions for increasing the amounts of rebaudioside A, resveratrol, and total phenolic substance in extracts obtained by the environmentally friendly ultrasonic probe-assisted extraction method were determined using Response Surface Methodology (RSM) and the accuracy of the obtained models were evaluated. The use of UPAE provided an important advantage in the recovery of bioactive compounds by enabling short extraction time, reduced solvent consumption, and effective mass transfer. The acoustic cavitation generated during sonication facilitated the disruption of plant cell walls and enhanced solvent penetration into the plant matrix, thereby improving the extraction efficiency of rebaudioside A, resveratrol, and phenolic compounds. As a result of the optimization, the ideal extraction conditions were determined as 7 min extraction time, 75 % ethanol ratio, 0.2 g plant amount, and 22 kHz ultrasonic frequency. Under these conditions, the predicted values of rebaudioside A, resveratrol, and TPC were calculated as 275.68 μg/mg, 5.61 μg/mg, and 55.33 mg GAE/g, respectively. The values obtained from experimental studies were calculated as 281.59 μg/mg, 5.74 μg/mg, and 54.16 mg GAE/g, respectively, and the findings from experimental applications were found to be consistent with these values (Table 4). These results are also in agreement with literature data demonstrating the effectiveness of different extraction techniques in obtaining valuable compounds from *Stevia rebaudiana* Bertoni leaves. For example, in a study using supercritical fluid extraction, the Reb-A content and total phenolic content were reported as 62.95 mg/g and 25.76 mg GAE/g, respectively [55]. In this study, the higher rebaudioside A and total phenolic content values obtained under RSM-optimized extraction conditions indicate that the applied approach was successful in improving the phytochemical content of stevia extracts.

**Table 4:** Predicted and experimental values of *Stevia rebaudiana* extract under optimum extraction condition.

	Response		
	TPC (mg GAE/g)	Rebaudioside A (µg/mg)	Resveratrol (µg/mg)
Predicted	55.33	275.68	5.61
Experimental	54.16	281.59	5.74

**Figure 5:**
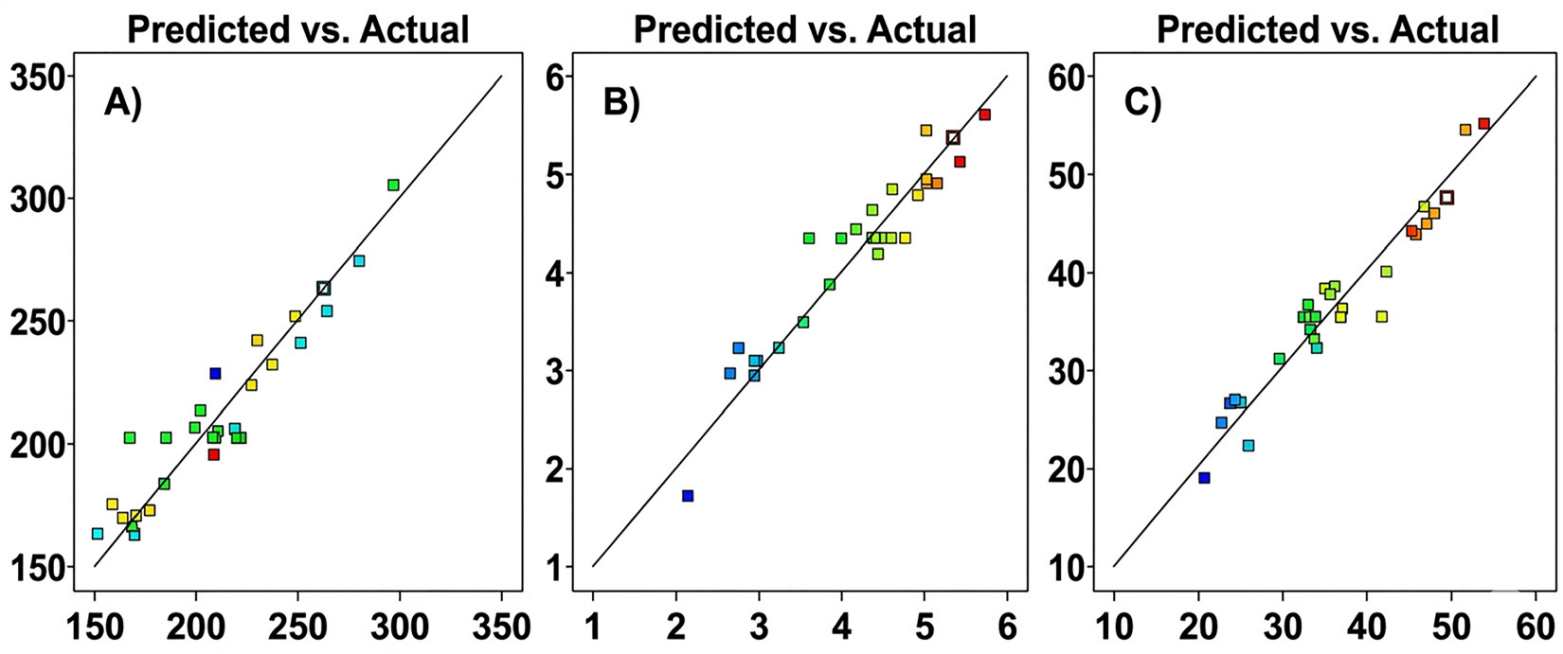
The correlation between the experimentally obtained versus the predicted values of the (A) rebaudioside A, (B) resveratrol, and (C) TPC of the *Stevia rebaudiana* Bertoni extract.

Figure 5 compares the model-predicted values with the actual values obtained experimentally. The linear regression curves plotted for rebaudioside A (A), resveratrol (B), and total phenolic content (TPC) (C) show a strong correlation between the predicted and experimental results. The distribution of most of the data points close to the diagonal line indicates that the model has an acceptable level of predictive ability. For rebaudioside A (Figure 5A), the predicted and actual values showed high agreement in the range of 150–300 μg/mg, with minimal deviations. For resveratrol (Figure 5B), model predictions and experimental findings were almost identical in the range of 2–6 μg/mg. For TPC (Figure 5C), a similar agreement was observed in the range of 20–55 mg GAE/g, with only minor deviations observed at a few points. These results demonstrate that the established second-order polynomial models are not only statistically significant fit and acceptable predictive capability within the experimental domain. The high *R* ^2^ values (Rebaudioside A: 0.90, Resveratrol: 0.91, TPC: 0.93) and low CV values (8–9% range) support the validity of the model, confirming that the model can accurately predict the yields of biologically active compounds in *Stevia rebaudiana* Bertoni extracts.

### 3.6 Estimation of total flavonoid content

Determining the total flavonoid content in *Stevia rebaudiana* Bertoni is essential due to its strong antioxidant, anti-inflammatory, and antimicrobial properties, which support its growing use as a natural antioxidant in food, health, and pharmaceutical industries. These bioactive compounds enhance Stevia’s value as an antioxidant supplement and food additive, emphasizing its therapeutic potential and industrial applicability. The total flavonoid content was determined by aluminium chloride colorimetric method. A calibration curve was constructed using rutin as the reference standard (*R* ^2^ = 0.9991), and the total flavonoid content under the optimized extraction conditions was calculated as 68.76 ± 0.33 mg RE/g of dry extract. In the study conducted by Khiraoui et al. [56], flavonoid contents in aqueous, ethanolic, and methanolic extracts of stevia leaves were shown to vary between 33.31 and 50.04 mg rutin/g, 19.87 and 33.86 mg/g, and 18.92 and 27.03 mg/g dry weight, respectively. Two different samples, organic and conventional stevia, were tested by Garcia-Mier et al. [57], and total flavonoid contents (TFC) were obtained as 0.17 and 0.19 mg RE/g dry samples, respectively. However, the results in this study were found to be higher than the reported TFC values.

### 3.7 Antioxidant assays

Antioxidants mitigate oxidative damage by scavenging reactive oxygen species (ROS), thereby reducing the risk of chronic diseases linked to oxidative stress, such as cancer, cardiovascular, and neurodegenerative disorders [58]. Several *in vitro* assays, including DPPH, FRAP, and CUPRAC, are widely employed to assess antioxidant capacity, each based on distinct chemical reactions that reflect different aspects of antioxidant behavior.

The percent inhibition values of *Stevia rebaudiana* Bertoni extract, l-ascorbic acid and trolox at different concentrations are given in Table 5. Additionally, the IC_50_ value, which represents the concentration of the sample required to reduce 50 % of the DPPH reactive oxygen species, was determined [59]. It is generally accepted that a lower IC_50_ value indicates higher radical scavenging activity [60]. The IC_50_ values were detected as 102.90 μg/mL, 4.69 μg/mL, and 5.44 μg/mL for *Stevia rebaudiana* Bertoni extract, l-Ascorbic acid and Trolox, respectively. In the literature, Ruiz-Ruiz et al. [61] determined the IC_50_ value for ethanolic *Stevia rebaudiana* Bertoni extract to be 335.94 μg/mL, whereas Shukla et al. [62] reported a value of 93.46 μg/mL. These findings reveal that the values ​​obtained in our study are generally consistent with the results reported in the literature.

**Table 5:** DPPH free radical scavenging activity of *Stevia rebaudiana* Bertoni, l-ascorbic acid and trolox.

	Concentration (µg/mL)	Inhibition DPPH % ± SD	IC_50_ (µg/mL)
*Stevia rebaudiana* Bertoni	375	95.41 ± 0.45	102.90
	250	92.70 ± 0.16	
	125	58.33 ± 0.16	
	50	29.92 ± 0.20	
	37.5	24.70 ± 0.45	
	25	19.09 ± 0.10	
	12.5	14.96 ± 0.10	
	5	11.36 ± 0.40	
	2.5	9.79 ± 0.65	
l-Acorbic acid	25	84.58 ± 0.71	4.69
	12.5	76.91 ± 0.98	
	5	53.19 ± 0.69	
	2.5	27.27 ± 0.35	
	1.25	13.94 ± 0.29	
	0.5	6.27 ± 0.79	
	0.25	3.27 ± 0.69	
	0.13	1.94 ± 0.59	
Trolox	25	90.97 ± 0.40	5.44
	12.5	90.28 ± 0.42	
	5	46.53 ± 0.77	
	2.5	25.73 ± 0.97	
	1.25	15.86 ± 0.56	
	0.5	9.58 ± 0.35	
	0.25	7.89 ± 0.92	
	0.13	6.57 ± 0.79	

In this study, the antioxidant activity of *Stevia rebaudiana* Bertoni extracts was determined using the FRAP method. A calibration curve was created using trolox as a standard (*R* ^2^ = 0.9998), and the antioxidant capacity was calculated as 529.75 ± 4.33 μmol TE/g. In the previous study conducted by Tadhani et al. [63], the total antioxidant capacity of *Stevia rebaudiana* Bertoni aqueous and methanolic extracts determined by the FRAP method was reported as 38.24 mg TE/g and 36.40 mg TE/g**,** respectively. Therefore, the antioxidant capacity of the extract obtained under the optimized extraction conditions in the present study was found to be significantly higher.

The total antioxidant capacities (TAC) of ethanolic extracts were analyzed with the CUPRAC method. TAC values ​​were calculated using the molar absorptivity (*ε*) of the standard reference compound trolox and were found to be 1.44 × 10^4^ L/mol cm. Also, the antioxidant results obtained with the CUPRAC method were evaluated by EC_50_ value that the extract concentration which equalled 0.5 of absorbance [64]. A lower EC_50_ value indicates higher antioxidant activity. The TAC values of the ethanolic extract at different concentrations and EC_50_ value are summarized in Table 6. In the study carried out by Chakma et al. [1], EC_50_ values were reported as 40 (µg/mL) for the shaker extraction method and 100 (µg/mL) for the Soxhlet extraction method. A study by Lremizi et al. [65] also demonstrated that different fractions of *Stevia rebaudiana* exhibited CUPRAC EC_50_ values ranging from 9.34 μg/mL (ethyl acetate fraction) to 392.50 μg/mL (essential oil). In the present study, the EC_50_ value was determined as 52.67 μg/mL, indicating a significant level of antioxidant capacity compared to previously reported data. Overall, these findings highlight that both the extraction technique and the solvent system used play a critical role in the antioxidant potential of *Stevia rebaudiana* extracts.

**Table 6:** Cupric ion reducing antioxidant capacity of *Stevia rebaudiana* Bertoni.

	Concentration (µg/mL)	TAC ± SD (mmol troloks/g)	EC_50_ (µg/mL)
*Stevia rebaudiana* Bertoni	122	0.66 ± 0.05	52.67
	60	0.65 ± 0.05	
	36	0.66 ± 0.07	
	30	0.66 ± 0.03	
	24	0.66 ± 0.06	
	18	0.65 ± 0.06	
	12	0.66 ± 0.08	
	6	0.65 ± 0.04	

## 4 Conclusions

In this study, an environmentally friendly ultrasonic probe-assisted extraction (UPAE) method was successfully developed and optimized to enhance the yields of rebaudioside A, resveratrol, and total phenolic content (TPC) from *Stevia rebaudiana* Bertoni. The integration of Response Surface Methodology (RSM) enabled the systematic evaluation and optimization of four key process variables (extraction time, ethanol concentration, plant material amount, and ultrasonic frequency) providing precise control over extraction efficiency. The optimized UPAE conditions (7 min extraction time, 75 % ethanol, 0.2 g plant material, and 22 kHz frequency) resulted in maximum yields of rebaudioside A (281.59 μg/mg), resveratrol (5.74 μg/mg), and total phenolic content (54.17 mg GAE/g), which were in close agreement with the model predictions, confirming the robustness and predictive capability of the RSM model. Additionally, the extraction yield was calculated under the optimized conditions and found to be 27.2 %.

Compared with conventional extraction techniques, the developed UPAE process significantly enhanced bioactive compound recovery while reducing solvent consumption and extraction time. The eco-friendly nature of the method was further strengthened by the use of ethanol as a green solvent and the implementation of a green HPLC quantification approach, eliminating the need for toxic organic modifiers such as methanol or acetonitrile. The high total flavonoid content obtained under the optimized extraction conditions further indicated that *Stevia rebaudiana* Bertoni is a rich source of flavonoid compounds. Antioxidant analyses (DPPH, FRAP, and CUPRAC) revealed that the resulting extract exhibited strong free radical scavenging and reducing properties, supporting its potential use as a natural antioxidant source. These findings demonstrate that the proposed UPAE–HPLC workflow provides a sustainable, scalable, and environmentally conscious platform for the extraction and quantification of high-value phytochemicals from *Stevia rebaudiana* Bertoni. Moreover, this study offers a valuable foundation for the development of green bioprocess technologies applicable to the food, nutraceutical, and pharmaceutical industries.
